# Automotive Application of Chemically Foamed rPET

**DOI:** 10.3390/polym17091251

**Published:** 2025-05-04

**Authors:** Veronika Anna Szabó, András Kovács, Sándor Kálmán Jakab, Tamara Zsuzsanna Böcz, Gábor Dogossy

**Affiliations:** 1 Department of Materials Science and Engineering, Audi Hungária Faculty of Vehicle Engineering, Széchenyi István University, Egyetem tér 1., H-9026 Győr, Hungary; jakab.sandor.kalman@sze.hu; 2 Institute of Materials Engineering, University of Pannonia, H-8201 Veszprém, Hungary; kovacs.andras@mk.uni-pannon.hu; 3 Department of Social Studies and Sociology, Apáczai Csere János Faculty of Humanities, Education and Social Sciences, Széchenyi István University, Liszt Ferenc u. 42, H-9022 Győr, Hungary; bocz.tamara@sze.hu; 4 Department of Vehicle Manufacturing and Engineering, Audi Hungária Faculty of Vehicle Engineering, Széchenyi István University, Egyetem tér 1., H-9024 Győr, Hungary; dogossy@ga.sze.hu

**Keywords:** recycled PET (rPET), hybrid foaming, flame retardancy

## Abstract

This study investigated the automotive applicability of parts produced from a newly developed foamed recycled polyethylene terephthalate (rPET). The injection molded part contained a combination of both endothermic and exothermic foaming agents and phosphorus (Exolit OP 1240) (OP)- and melamine polyphosphate (MPP)-based flame retardant agents. The parts were produced using a breathing mold technique to achieve a suitable level of foaming. The aim was to produce lighter parts made of recycled material that also complied with the fire safety automotive industry standards. Computer tomographic scans revealed the foam structure formed successfully, which contributed to an improved strength-to-weight ratio. The scans further showcased that larger cells tended to form in the thicker areas within the part, while smaller cells generally formed in the thinner areas. Finite element simulations showed that the large cell formation in the thicker parts had no effect on the part’s load bearing property, and there were not stress concentration points after the boundary conditions were defined. The sample produced from the material was determined to be a possible replacement of small-sized automotive components.

## 1. Introduction

Plastic production has globally grown at a drastic rate over the past decades as production reached 400.3 million tonnes by 2022, which equates to a 1.6% increase from the previous year [1]. Polyethylene terephthalate (PET) is one of the most widely used plastics, particularly within the packaging industry [2]. Its mechanical properties have several advantages as it is mainly used because of its high abrasion and impact resistance properties [3]. Contrarily, PET’s degradation time of half a millennium poses serious environmental challenges to society [4]. The slow increase in recycling rates is not sufficient and urges the need to develop more efficient recycling technologies [5]. PET waste continues to rise within the marine ecosystems and raises concerns not just for flora and fauna but for humanity as well [6].

One of the biggest technological obstacles in the usage of recycled PET (rPET) is chain breakage, that is, the weakness of the material, which restricts the applicability of the chemical foaming processes. Chain breakage restoration and the optimization of the mechanical properties of the foam structure are essential for the successful industrial application of rPET materials. Unsuccessful foaming impacts not only the quality of the final product but also the processability and stability of the manufacturing process as the disruption of the molecular chains can lead to significant deterioration in the properties of the final product. Mechanical strength and cell structure uniformity are essential in the production of foamed products. In their research, Bocz and colleagues showcased that the use of the CESA Extend chain extender can effectively reduce chain breakage, optimize the molecular chain length, and propagate foaming [7].

In the initial stages of our research, we also aimed to improve the mechanical properties of the foam structure, by adding 10% impact modifier to the mixture, which resulted in an 18.48% impact strength improvement. This result highlights the importance of modifiers in enhancing the mechanical stability of the foam structure. Our results showed that chemical foaming agents could be successfully implemented in the production of rPET specimens with closed-cell foam structure, which not only had favorable mechanical properties but were also industrially viable [8,9].

In a previous phase of our material development, we investigated the effect of endotherm–exotherm hybrid foaming on the mechanical and morphological properties of rPET foams. This research revealed that the hybrid foaming process significantly improved the cell structure homogeneity and increased cell numbers, which contributed to the reduction in the foam density. This reduction in density is crucial for producing lighter yet mechanically stable foams. In contrast, when only an endothermic foaming agent was used, the samples showed a less homogeneous cell structure [10].

Jinfu Xing et al. [11] investigated how the mechanical properties of three thermoplastic polyurethanes (TPUs) with different hardnesses changed when adding chemical blowing agents and using the breathing mold technique. They found that the tensile strength decreased drastically, by at least 25%, upon foaming; however, the thermoplastic polyurethane with the highest hardness had the best mechanical properties when compared with the ones with lower hardness. The tensile strength was measured at 13.59 MPa compared with 8.53 MPa and 11.79 MPa for the other two materials, while the flexural strength was 15.46 MPa for the hardest TPU; meanwhile, the others produced 8.82 and 5.42 MPa during their measurements.

Steven Mendoza-Cedeno and colleagues [12] experimented with four different polypropylenes with different molecular weights using a chemical blowing agent during the injection molding. According to their results, polypropylenes with higher molecular weight expanded better and formed a stronger cell structure, while the ones with low molecular weight formed a denser cell structure. Furthermore, with lower molecular weight, the outer shell was thinner, and cell formation began sooner.

When examining the addition of exothermic foaming agent to the formula even at the lowest (0.5%) concentration, the addition significantly improved the uniformity of cell distribution, compared with the samples that only contained the endothermic foaming agent. At 1% concentration of the exothermic foaming agent, an increase in porosity was observed without any significant negative effect on the mechanical properties. However, at higher concentrations the exothermic foaming agent had negative effects on the cell structure and the porosity as well. Our research showed that higher porosity alone did not necessarily weaken the mechanical resistance of the foamed material as long as the cell distribution remained homogeneous. With 3% endotherm and 1% exotherm foaming agent concentration levels, a 16.6% porosity level (highest of all samples) was achieved, while the sample also possessed great mechanical properties: Young’s modulus was measured at 1.49 ± 0.1 GPa, the maximum tensile stress was 28.94 ± 1.33 MPa, and the Charpy impact strength was 3.51 ± 0.46 kJ/m^2^. Based on these results, this ratio was selected to continue our research [13].

The production of hybrid foamed rPET samples containing different flame retardant additives was tested based on the previous results, with the primary objective of maintaining the mechanical strength of the foam while achieving a V0 rating in accordance with the UL94 standard. Our tests showed that combinations of flame retardants, in particular the Exolit AP 422 (AP) and the Exolit OP 1240 (OP), had notable effects on foam homogeneity and mechanical properties. The addition AP flame retardant resulted in higher porosity and a more uneven cell structure, which had a negative effect on mechanical stability and fire retardancy. In contrast, the use of OP resulted in smaller cell sizes and lower porosity, thus achieving better results in terms of mechanical resistance and fire retardancy [14].

During the research, we paid particular attention to the addition of MPP (melamine polyphosphate) as it synergized with both the AP and the OP flame retardants. MPP improved the stability of the cell structure and reduced the cell size, which, in combination with both flame retardants, resulted in significant mechanical and fire retardancy improvements.

The European Union’s End-of-Life Vehicles Directive (2000/53/EC) plays a major role in promoting sustainability in the automotive industry by introducing strict rules for the treatment and recycling of end-of-life vehicles. The directive aims to reduce the number of vehicles going to landfills and to encourage recycling and reuse. The regulation requires that at least 85% of vehicles are recycled, which is a major challenge for the industry [15]. The use of recycled materials is environmentally beneficial, reduces raw material consumption, and is cost-effective. In addition, the use of recycled materials contributes to reducing the ecological footprint of car manufacturing and helps manufacturers to comply with policies [16].

rPET foams particularly could be promising for the automotive industry as they are lightweight, have good mechanical properties with adequate fire resistance and they can be used to manufacture components that play a key role in meeting the structural and safety requirements of vehicles.

The work of Jiang et al. [17] aimed to create lightweight and durable structures using recycled materials with the focus on mechanical strength. The core of the composite is made from recycled PET foam combined with polypropylene (PP), reinforced with outer layers of flax fiber. In the manufacturing process, the composite was produced by high-pressure compression molding, and thermoplastic adhesive films were used to bond the rPET foam core and the flax fiber/PP layers, resulting in a strong yet lightweight structure, deemed sustainable and comparable to the regular materials used based on its mechanical properties. Its flexural strength was around 50 MPa, while its density was only 0.25 g/cm^3^. This also displayed high impact resistance, which can be suitable for automotive applications where there is high stress concentration on the parts, such as the side panels of trucks.

Bedell et al. [18] used polyester polyol from recycled PET to make flexible polyurethane foam. This polyurethane foam is used to make automotive interior trim panels. The researchers used polyester polyols made from recycled PET waste to synthesize flexible polyurethane (PU) foams, designed for automotive interior component production. In their research, they tested five different formulations wherein up to 50% of the petroleum-based polyol content was replaced with recycled polyols. The foams were subjected to mechanical, thermal, morphological, and physical tests to assess their potential for industry application for car interiors. With the foams containing 50% rPET, Young’s modulus increased by 121%, tensile strength by 67%, tear resistance by 32%, and compressive modulus by 150% compared with petroleum-based formulations. This indicates that the usage of recycled materials not only can be beneficial to increase sustainability but can also serve as an improved alternative to the existing materials used [18].

In this paper we investigated the automotive applicability of the two best performing foamed rPET samples from our previous research. In our experiments, we produced side window shading roller holders found in buses. This part was originally produced through injection molding made from polyamide 6 with 15 wt.% glass fiber content (PA6GF15). However, with our newly developed foamed material, we aimed to reduce its weight, prove the potential of automotive applicability of foamed materials, and promote the usage of recycled materials. During testing the emphasis was on ensuring that the material met the mechanical and fire retardancy industry requirements.

## 2. Materials and Methods

### 2.1. Materials

In this research, our self-developed foamed material [19] was used; its composition is depicted in Table 1. The blue crystallized recycled PET (rPET) used in the research was provided by Fehérvári Group Zrt. (Budapest, Hungary) (inherent viscosity (IV) of 0.8 dL/g) in granulated form. The CE-SA Extend NCA0025531-ZA chain-extender additive used was supplied by Clariant (Muttenz, Switzerland), which contained the Joncryl ADR 4368 epoxy-based styrenerol-acrylic multi-functional oligomeric reagent. Dupont Elvaloy PTW (Midland, MI, USA) was used as an impact modifier to improve the mechanical resistance of the samples. One of the foaming agents used was the Tracel IM 7200 endothermic compound, supplied by Tramaco (Tornesch, Germany), with a gas expansion rate of 120 mL/g, a blowing agent content of 70%, and a decomposition temperature of 220 °C. The other foaming agent used was the Tracel IM 3170 MS (Tornesch, Germany) exothermic foaming agent, with a 50 mL/g gas expansion rate and a decomposition temperature of 170 °C. One of the flame retardants was the Exolit OP 1240 (OP), which is a halogen-free flame retardant additive containing phosphorus, supplied by Clariant (Muttenz, Switzerland). Clariant has a decomposition temperature of approximately 350 °C, a 6.0 PH value, and a water solubility of 0.1 g/100 mL. The additive’s particle size distribution was 20 µm, which allowed a homogeneous distribution in plastics. The used MPP was the Budenheim Budit 342 (Budenheim, Germany), a halogen-free melamine phosphate-based flame retardant. It had a phosphorus pentoxide content of approximately 30% and a nitrogen (N) content of 43%. Its decomposition temperature was estimated at 325 °C.

### 2.2. Methods

In our previous study [19] we produced dumbbell specimens, where the mechanical properties of the material compositions were analyzed. The tensile strength tests were carried out using an INSTRON 5582 (Norwood, MA, USA) universal testing machine with a grip length of 100 mm. The test speed was set at 1 mm/min to determine the elastic modulus and at 5 mm/min for measuring the tensile strength in accordance to the ISO 527-2 standard [20]. The maximum flexural stress and flexural modulus value following the guidelines of the ISO 179 standard [21] were measured, using the same machine, and flexural tests were conducted with a crosshead speed of 5 mm/min and a support span of 64 mm. The impact resistance was measured using a CEAST 6545 impact tester (Ceast, Torino, Italy) with a 2 J hammer and a 62 mm span length [22]. In our previous research, we also performed a UL 94 fire safety test on the foamed samples to determine their flame retardancy performance. During the testing of the 4 × 10 mm cross-sections, the self-extinguishing time and flame spread rates of the test samples were measured in two different orientations in accordance with the UL-94 harmonized test procedure [23,24].

Thermogravimetric analyses (TGAs) were performed on the materials to determine the decomposition rate. The thermogravimetric analysis (TGA) was performed using a TA Q5000 instrument (TA Instruments LLC, New Castle, NH, USA), programmed to heat from 30 °C to 800 °C at a rate of 20 °C per minute under a nitrogen atmosphere. The onset temperature (T_onset_), 50% decomposition temperature (T_-50%_), decomposition speed (dTG_max_), and char yield were read from the curves.

The holders were produced using the Arburg Allrounder Advance 420C Golden Edition (Arburg, Loßburg, Germany) injection molding machine (its parameters are listed in Table 2). To achieve a suitable foaming level, the breathing mold technique [25] was utilized. During this process, the polymer mixed with the foaming agent was injected into the mold cavity, where a solid surface layer of the part was formed during cooling. Subsequently, the mold was minimally opened, and the space increased while the pressure decreased. While the specimen still cooled down in the partially (0.5 mm) opened mold, within its structure, foaming still occurred.

The internal structure of the brackets was examined using industrial computed tomography (CT) equipment. The average scan time was 24 min as 1500 projections images were captured in two frames per projection mode. The voltage and current used during scanning were 165 kV and 65 microamps, respectively. The VG Studio Max 2022/2220 software pack was used during analysis and visualization. The CT scan generated a detailed 3D image of the sample, allowing for the identification and measurement of the cell structures within. By subtracting the volume of these cells from the total volume of the sample, the remaining solid material’s volume was obtained. The sample’s density was then calculated by dividing the mass of the material by this adjusted volume, providing a precise assessment that reflected the presence of internal porosity.

With this value the deformation of the produced parts upon applied load was simulated, to determine whether it could be used as a replacement for the previously used material. The VG Studio 2023/1 Structural Mechanics finite element simulation algorithm was used, and the CT scans allowed us to study the full geometry of the product. A linear elastic material model was used, for which the Poisson ratio was 0.3053, determined based on the tensile tests of the dumbbell specimens. The system included the results of the porosity analysis during the calculations of the results. During the real structure analysis, the cell size was 8 voxels, which equaled the resolution from the porosity analysis. The aim of this was to find the critical cross-section of the part. We wanted to determine the critical load location within the integral foam products of varied structure densities and where and what types of cells could be found at these points. For the simulation, a 100 N horizontal force was applied to the part, while the back panel and the two screw locations were fixed, to map out the load distribution and draw out tensile lines.

## 3. Material Properties

This section provides a brief insight into the material properties used for producing the holders, referring to our previous study [17].

### 3.1. Mechanical Tests

**Figure 1:**
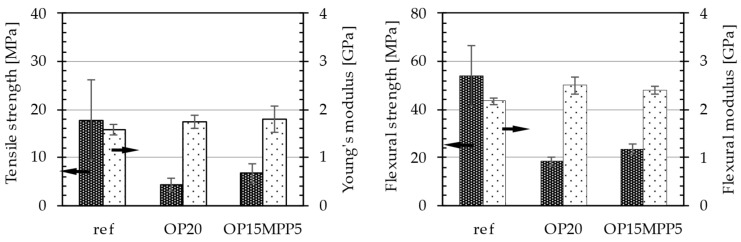
Tensile properties and flexural properties of the samples. Left side columns indicated by left arrow displays data for the left axis, Right side column indicated by the right arrow displays data for the right axis.

The materials were analyzed to determine their mechanical properties and how the different flame retardants affected them, presented in Figure 1.

Based on the comparison between OP20 and OP15MPP5 samples, the addition of MPP (melamine phosphate-based flame retardant) had lesser negative effects on the mechanical properties. The tensile strength of the OP15MPP5 sample was higher (6.66 MPa) compared with the OP20 sample (4.24 MPa), suggesting that MPP contributed to the higher mechanical strength of the material, while the flame retardant had a negative effect on the tensile strength. There was a slight improvement in the Young’s modulus, with the OP15MPP5 sample (1.79 GPa) being slightly higher than the OP20 sample (1.73 GPa). It is notable that results here also improved compared with the reference value. Similar results were seen in the bending properties. There was a large drop in flexural strength compared with the reference sample, but the flexural modulus values were improved with the addition of the flame retardant. This suggests that the OP15MPP5 sample had the better mechanical property between the two, and its higher rigidity compared with the reference could be an advantage in automotive applications.

The results of Charpy’s impact test also provide interesting observations between OP20 and OP15MPP5 samples. The reference had an impact strength value of 2.26 KJ/m^2^, while for the OP20, 0.04 KJ/m^2^ was measured. In contrast, the impact resistance of OP15MPP5 sample was 0.64 kJ/m^2^, a significant improvement compared with the OP20 sample. Although the value was relatively low, the increase suggested that the addition of MPP improved the impact resistance of the material.

The differences between the two samples’ mechanical properties suggested that the microstructure of the materials and the used additives had a significant impact on foam structure.

### 3.2. Flammability

**Figure 2:**
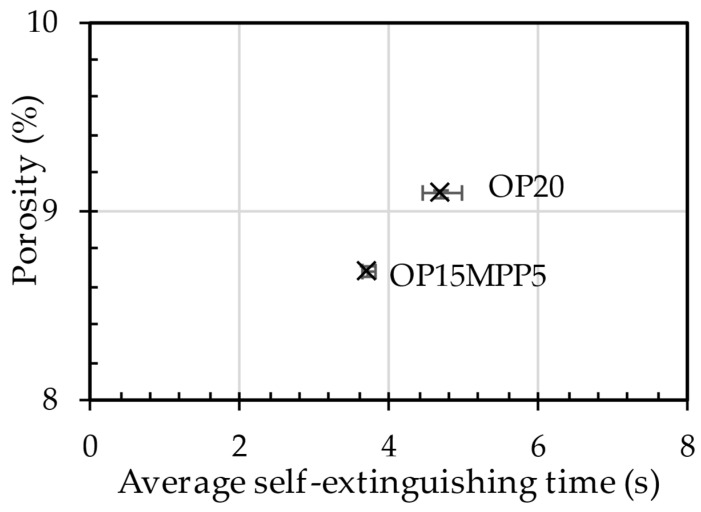
Self-extinguishing time as a function of porosity.

In Figure 2 the self-extinguishing time in relation to the porosity can be seen. From the results, we see that the addition of MPP reduced porosity. For the OP20 sample, the higher porosity and the longer self-extinguishing time allowed the sample to meet the criteria for the V0 flammability category. More internal cells made the material more prone to flame propagation as the gas bubbles created during foaming were easily heated by the gas traps of nitrogen (N_2_), carbon monoxide (CO), hydrogen (H_2_), and ammonia (NH_3_), depending on the foaming agent. Nitrogen alone did not promote the spread of the flames; therefore, the others promoted combustion and inhibited faster self-extinguishing. This also explained the longer self-extinguishing time of the OP20 sample.

### 3.3. TGA

A summary table of the thermalgravimetric analyses can be seen in Table 3. The reference sample had a higher onset temperature than the samples mixed with flame retardant; however, they almost fully disintegrated with a 3.8% char yield. The OP15MPP5 sample started decomposing early, at 371 °C, and showed the slowest decomposition speed (1.39%/°C) with 50% decomposition measured at 458 °C. This was a significant improvement, showcasing good thermal stability and signaling good charring properties as 23.1% remained after the end of test. The OP20 samples showed a decrease in disintegration speed compared with the reference, with 1.59%/°C, but the onset temperature and the 50% decomposition mark were close to the values of the reference. However, with 22.1% char yield, its flame retardancy was unquestionable, and based on the OP15MPP5 results, the combination of flame retardants seems promising.

## 4. Applicability Testing

The aim of the experiment was to produce an automotive component utilizing the material composition and technology we developed. The chosen components were the side window shading roller holders, used in Daimler Buses GmbH, Leinfelden-Echterdingen, Germany (Mercedes-Benz Group, Stuttgart-Untertürkheim, Germany) buses (formerly Evobus GmbH, Leinfelden-Echterdingen, Germany). These were previously produced from polyamide 6 with 15 wt.% glass fiber content (PA6GF15) and a density of 1.23 g/cm^3^ [26], which was replaced by our self-developed rPET foam. The holder element was necessary to ensure that the shaders were securely fixed while also ensuring smooth and easy rolling of the side window shader upon pulling, while the part remained durable to ensure its longevity.

### 4.1. CT Scans of the Automotive Component

**Figure 3:**
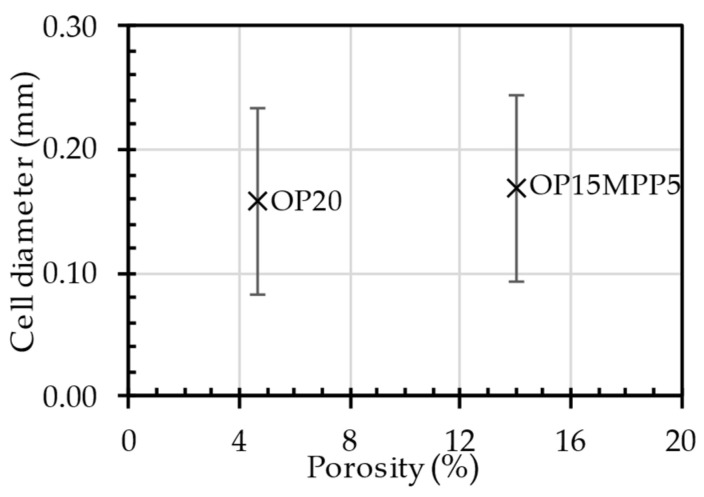
The porosity of the samples produced as a function of the cell dimensions.

**Figure 4:**
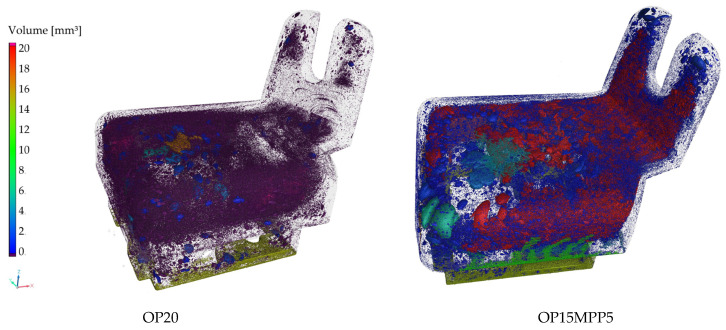
CT scan of the manufactured samples.

The differences in porosity and cell diameter between the two samples chosen for this experiment are shown in Figure 3. A three-dimensional image of the components is shown in Figure 4. Significant differences between the porosity of the OP20 and the OP15MPP5 samples were visible, where the porosity values for the OP20 sample were lower than the porosity values of the OP15MPP5 sample. The OP20 sample had smaller, less connected cells, while the OP15MPP5 had larger cells. However, the results were skewed by the largest cells. The overpressure in the immediate vicinity of the firing point due to the small cross-sections did not result in larger cell sizes. For the sample OP15MPP5, there were nine large cells with cell diameters several times larger than the average; however, since there were 283,828 cells overall in the test range, this had a smaller impact on the average cell size but a large impact on the porosity. Javier Gómez Monterde, in his doctoral thesis, observed similar behaviors, where the formation of similar structures was due to the formation of cells during and after injection [27]. The addition of MPP helped the foaming agent to form the cell structure in all cross-sections. It also affected the cell density as similarly, we compared the same 1 mm^3^ volume, which had 23.8 cells per 1 mm^3^, with 34.2 cells per 1 mm^3^ in the OP20 sample, where only 1 out of nearly 50,000 cells had exceptionally high cell volume. This left room for numerous small cells instead of a few elongated and collapsed ones. However, the porosity of the sample was much lower, only a third of the OP15MPP5 samples. The small cells in sample OP20 covered a large surface area in all cross-sections of the sample but did not show a deeper, more comprehensive cell structure, in contrast to the tighter cell structure observed in sample OP15MPP5, which can be seen on the CT scans. The addition of MPP increased the porosity without significantly affecting the average cell size, indicating that the change in viscosity of the material positively affected the foamability, while the closed-cell foam structure remained intact with a couple of exceptions.

The integral foam structure is distinctly similar to the structure of human bones, in particular their trabecular structure, which is an open-cell, sponge-like material. The trabecular structure in the bone has excellent mechanical properties as its low density provides high strength and load-bearing capacity while minimizing mass, which is important for load-bearing function. Likewise, integral foams combine a dense outer layer and a lightweight, porous inner section, which allows the material to have optimal mechanical properties, including high energy absorption and stress distribution, while maintaining their light weight. This structure is ideal where the strength-to-weight ratio is a critical factor in applicability [28].

**Figure 5:**
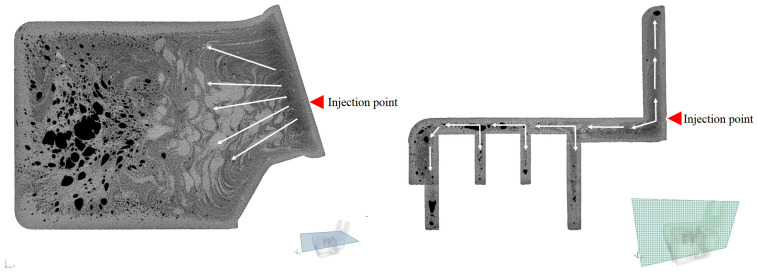
Melt flow in the holder made with 20 phr OP, indicated by arrows.

**Figure 6:**
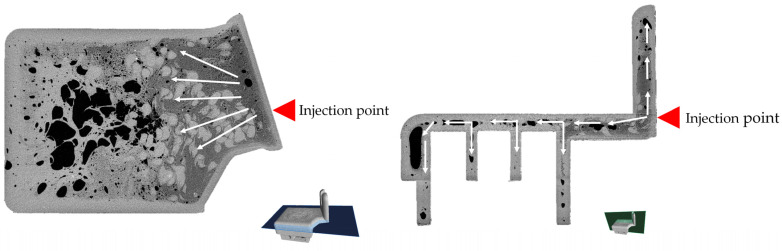
Melt flow in the holder made with 15 phr OP and 5 phr MPP, indicated by arrows.

The direction of the plastic flow is apparent in the images (Figure 5 and Figure 6). From the point of injection, flow started from the right side and spread out from there. The foaming agent had the possibility to expand the gas bubbles on the left side of the product due to the use of the breathing mold technology on the larger volume. The resulting increased cell density is shown on the left side of the image. On the right side, smoother, denser material with fewer cells is visible, and no cells were created in the light parts of the image. It is clearly shown in the samples that injection molding with chemical foaming produced a perfect integral foam. The mixture, still in a fluid state on the inside, allowed the foaming agent to expand the gas bubbles at the nodal points and create the closed cell structure shown on the left image. Due to the lower decomposition temperature, the exothermic foaming agent first created nodal points in the middle of the unit at a lower pressure, which were joined by the endothermic foaming agent and where the gas bubbles were most likely to expand when the mold was opened. The significant contrast between cell sizes and cell distribution perfectly illustrates the pressure variation. Initially, the high pressure promoted flow, but as the pressure decreased, foaming took place, and foam with varied porosity was formed at the different cross-sections of the product. This is critical in the automotive or industrial applications as it maintains structural integrity while keeping the part lightweight.

Accurate management of the pressure profile was key to optimizing the foam morphology [29], striking a balance between too much pressure (which suppresses foam formation) and too little pressure (which can lead to incomplete filling or uneven foam structure). This pressure gradient allowed the material to foam successfully in the lower-pressure sections.

### 4.2. Testing the Applicability of an Automotive Product Using Finite Element Simulation

**Figure 7:**
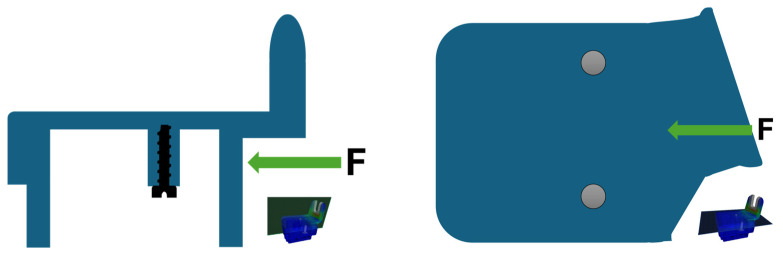
The schematic diagram of the simulation.

As a continuation of our previous research, finite element analysis (FEA) was used to determine the applicability of the produced part. The aim was to determine whether the part produced from foamed rPET could be a suitable replacement to the regular bus component while also reducing the component’s weight. It was expected that the bracket would fail at the area with the densest cell structure. The schematic diagram of the simulation is depicted in Figure 7. The 100 N horizontal force applied to the part is indicated by the green arrow, while the screw and the grey circles the location of the screw fixing the part.

**Figure 8:**
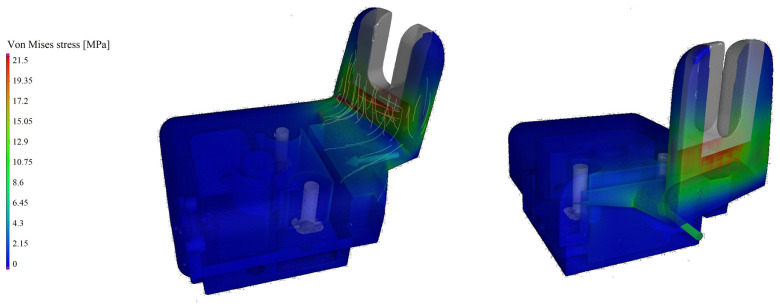
Simulation test result.

The simulation is depicted in Figure 8, has concluded that the highest stress concentration points were near the joints between the back panel and the other structural parts (indicated by the red area). It is important to mention that the porous area in question, where the construction is weaker, in practice does not carry considerable load, therefore the part is usable from a functional standpoint. We examined the CT scans of sample OP15MPP5, and the 10 largest cells are not located on the junctional surface. The largest cell here was (referring to the porosity image displayed on Figure 2) 36% larger than the average; therefore, we assumed that the product’s thinning did not preclude the suitability of this blend for the automotive industry. This simulative approach allowed us to obtain a reliable foundation for the structural load distribution, and it was concluded that the application of foam structure for the fixture does not prevent the utilization in the car industry. During the design of the mounting part, the assurance of mechanical stability is essential, which can be ensured with the compound we used.

Based on the CT results, an overall volume of 12.879 cm^3^ was determined for the bracket. When the PA6GF15 material was used, the product’s density was 1.23 g/cm^3^, and the weight was approximately 15.84 g. With the self-developed foamed material, the weight was reduced to 15.19 g, and a 4.8% density decrease was achieved, while the structural properties and mechanical load bearing capability were maintained, allowing the replacement of the original material with this foamed rPET composition for the production of this part.

## 5. Conclusions

This in depth study investigated a possible replacement of an automotive part made from polymeric material. The goal was to replace the previously used polyamide 6 polymer mixed with 15 wt.% glass fiber (PA6GF15) with a composition of foamed recycled polyethylene terephthalate (rPET) with added flame retardant agents. The part in question was successfully manufactured with improved flame retardancy and reduced density while maintaining structural integrity and load-bearing properties. The CT analysis of the molded automotive component made from the rPET integral foam confirmed that the developed product could be suitable for other automotive applications. As it provides an excellent strength-to-weight ratio with a 5% decrease in density, this is an important factor in the weight reduction of automotives. Based on the finite element simulations, we can conclude that the mechanical stability of the foamed rPET material remained satisfactory. In the OP20 sample at the junction area between the back sheet and the cover sheet, cells were scarcely found. In contrast, the OP15MPP5 sample exhibited a high cell density, yet the formed cells were of smaller diameter. Thus, despite the increased cell density, the structure remained suitable only for resisting lower forces. The homogenous distribution of OP and MPP compounds contributed to even the growth of the foamed cell, and the usage of foamed rPET in the production of other automotive parts is possible and its investigation worth pursuing.

## Figures and Tables

**Table 1:** Composition of the tested samples (unit: parts per hundred resin (phr)).

	rPET	Calriant CESA Extend	Du-Pont Elovaloy PTW	Tracel IM 7200	Tracel IM 3170 MS	Clariant Exolit OP 1240	Budenheim Budit 342
OP20	100	2	10	3	1	20	0
OP15MPP5	100	2	10	3	1	15	5

**Table 2:** Injection molding parameters.

Description	Unit	Value
Clamping force	kN	150
Nozzle temperature	°C	260
Injection pressure	bar	650
Injection speed	cm^3^/s	30
Holding pressure	bar	150–50–20
Holding pressure time	s	2–1
Intermediate mold opening	mm	0.5
Residual cooling after mold opening	s	20
Mold temperature	°C	35

**Table 3:** TGA analysis results of the foamed polymers mixed with flame retardants.

	T_onset_ [%]	T_-50%_ [%]	dTG_max_ [%/°C]	Char Yield [%]
ref	410	469	1.93	3.8
OP20	401	464	1.59	22.1
OP15MPP5	371	458	1.39	23.1

## Data Availability

Data are contained within the article.
